# Shrews (Soricidae) of the lowland forests around Kisangani (DR Congo)

**DOI:** 10.3897/BDJ.7.e46948

**Published:** 2019-12-20

**Authors:** Frederik Van de Perre, Herwig Leirs, Julien Cigar, Sylvestre Gambalemoke Mbalitini, Jean-Claude Mukinzi Itoka, Erik Verheyen

**Affiliations:** 1 Evolutionary Ecology Group, University of Antwerp, Antwerp, Belgium Evolutionary Ecology Group, University of Antwerp Antwerp Belgium; 2 Belgian Biodiversity Platform, Brussels, Belgium Belgian Biodiversity Platform Brussels Belgium; 3 Centre de Surveillance de la Biodiversité, Kisangani, Democratic Republic of the Congo Centre de Surveillance de la Biodiversité Kisangani Democratic Republic of the Congo; 4 Faculté des Sciences, UNIKIS, Kisangani, Democratic Republic of the Congo Faculté des Sciences, UNIKIS Kisangani Democratic Republic of the Congo; 5 Royal Belgian Institute of Natural Sciences, Brussels, Belgium Royal Belgian Institute of Natural Sciences Brussels Belgium

**Keywords:** pitfall, removal trapping, tropical lowland forest, Democratic Republic Congo, Soricidae

## Abstract

**Background:**

The Congo Basin rainforest is the second largest rainforest in the world and one of the most biodiverse regions on Earth. Nevertheless, the Congo Basin biodiversity remains to be fully mapped, with many species awaiting discovery or official description. In recent years, much effort has been put into research on shrews (Soricidae), particularly in the region around Kisangani (D.R. Congo). Shrews are opportunistic feeders that are able to forage on a large diversity of invertebrate prey and therefore play an important role in the forest ecosystem. Furthermore, as they largely depend on forest habitats and have limited dispersal capacities, shrews form an interesting model group to study biogeographic patterns in the Congo Basin.

**New information:**

This paper collates the efforts on shrew research from the wider region around Kisangani, in the centre of the Congo Basin. Apart from sampling information, the dataset includes morphological measures, DNA sequences and photographs. This dataset is therefore critical in the study of the taxonomy and ecology of Soricidae in the Congo Basin lowland rainforests.

## Introduction

The Congo basin rainforest is the second largest in the world and one of the most biodiverse regions on earth (Mittermeier et al. 2003, Lewis 2005). Both the forest and its biodiversity are threatened by forest loss and bushmeat hunting (Malhi et al. 2013). Despite its importance for climate change mitigation and biodiversity conservation, knowledge on the impact of forest loss and degradation and bushmeat hunting on local biodiversity is currently lacking (Gibson et al. 2011, Alroy 2017, Phillips et al. 2017). In fact, the biodiversity of the Congo basin is generally understudied, with several new species being discovered every year (e.g. Colyn et al. 2010, Stanley et al. 2013).

Our knowledge on occurrence, ecology and taxonomy of shrews (Soricidae) in the Congo basin is currently incomplete (Mukinzi et al. 2005, Gambalemoke 2014, Jacquet et al. 2015). Shrews represent critical food web links via their role as predators of small vertebrates and invertebrates and as prey for several vertebrate predators (Churchfield et al. 2004). Apart from their importance in the ecosystem, shrews are also a suitable model taxon for evaluating biogeographic and historical hypotheses (Quérouil et al. 2003). Indeed, the central Congo basin (i.e. the lowland forests south of the Congo river) harbours less terrestrial vertebrate species than the northern part which is due to the smaller habitat area and isolated position of the central Congolian lowland forests (Van de Perre et al. 2019). As richness is limited by habitat area and isolation, this implies that diversity differences amongst Congolian lowland forests are mostly due to forest-associated taxa with limited dispersal capacities, such as shrews.

Therefore, this paper assembles shrew occurrences from three studies in the central Congo Basin (Gambalemoke et al. 2008b, Mukinzi 2014, Van de Perre et al. 2018) and includes the metadata of the captured specimens (morphological measurements, DNA and sampling details) which allows for the advancement of the knowledge on taxonomy and ecology of shrews in the central Congo basin.

## General description

### Purpose

This paper assembles data collected in the framework of the PhD theses of Jean-Claude Mukinzi Itoka, Sylvestre Gambalemoke Mbalitini and Frederik Van de Perre. These three theses were executed at or in collaboration with the University of Kisangani, the University of Antwerp and the Royal Belgian Institute for Natural Sciences. Although the design and purpose of the three studies differ, the sampling design (the method in which shrews were collected) is equal across studies, which justifies the publication of the dataset as a whole.

## Project description

### Study area description

**Figure 1.**
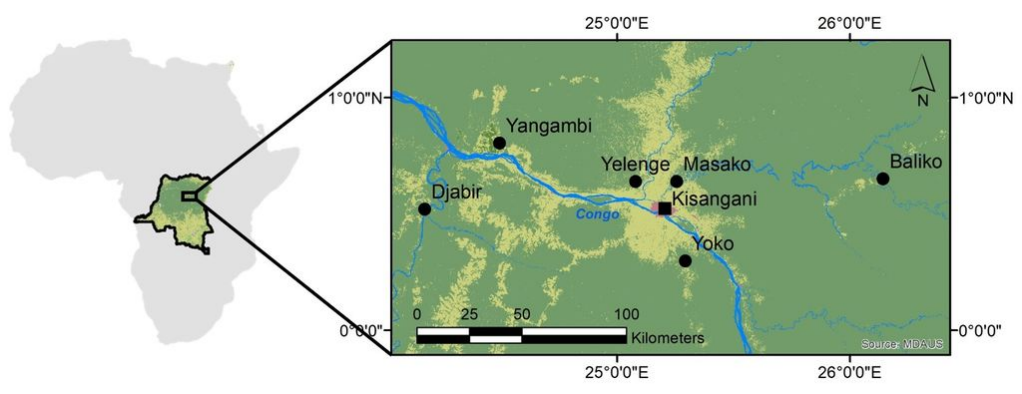
Sampling localities (dots) in the environs of Kisangani (see Table 1 for additional details). The city of Kisangani (square) is surrounded by a mosaic of agricultural land and regrowth forest (light green), while old-growth forests (dark green) can be found throughout the area. Blue lines represent the Congo River and its tributaries. The map on the left shows the situation of the study area within Africa.

We compiled data from three studies in the region around Kisangani (Gambalemoke et al. 2008b, Mukinzi 2014, Van de Perre et al. 2018). The combined data represent 36 sampling sites in which sampling effort was equal and distributed within 6 localities in the Tshopo Province (Fig. 1 and Table 1). Sampling localities are separated by the Congo River and some of its major tributaries (Tshopo, Lindi, Lomami).

In the study area, forest disturbance is mainly in the form of slash-and-burn agricultural activities, followed by abandonment and secondary succession. Fallow land, the pioneer stage of forest recolonisation, contains dense thickets with few tall trees. Regrowth forests generally are dominated by *Musanga cecropioides* in the canopy. Old-growth, closed canopy forests represent a range of vegetation, including mixed, semi-deciduous forest, monodominant forest of *Gilbertiodendron dewevrei* (De Wild.) J. Leonard and monodominant forest of *Brachystegia laurentii* (De Wild.) Hoyle. In some localities, sampling was also conducted in abandoned oil palm plantations. Apart from Yangambi (Van de Perre et al. 2018), quantitative data on tree composition of each sampling site is lacking.

Following the revised Köppen-Geiger classification (Peel et al. 2007), the climate of the region is Af-type tropical rainforest climate. At the Yangambi meteorological station, the annual precipitation is 1839.5 ± 205.7 mm (1980–2012) and average dry season length is 3.3 ± 1.3 months (a month is dry if it receives less than 100 mm of precipitation). Dry seasons occur in December–February and June-August. Temperatures are high and constant throughout the year, with a minimum of 24.2 ± 0.4°C in July and a maximum of 25.5 ± 0.6°C in March (Doetterl et al. 2015).

### Funding

F.V.d.P. was supported by a Ph.D. fellowship from the Research Foundation – Flanders and by the Belgian Science Policy Office (COBIMFO Project; Congo Basin integrated monitoring for forest carbon mitigation and biodiversity; contract no. SD/AR/01A).

## Sampling methods

### Sampling description

In all localities, shrews were sampled using the paceline method, which involved placing 20 pitfall traps at 5 m intervals on transects (Nicolas et al. 2003). Pitfall traps consisted of non-baited buckets (10-litre, 30×30×23 cm) that were buried in the ground, with rims even with the ground surface. A plastic drift fence (100 m) was set to increase capture effectiveness by guiding shrews toward traps. Pitfall traps were maintained at their locations for 21 days and were checked daily. Only in Yoko, these pitfall lines were set for multiple sessions at the same location. In all other locations, trapping was only conducted once (Table 1).

### Quality control

Species were identified based on external morphology and cranio-dental characteristics. In addition, species assignments were confirmed for several specimens of each species by molecular analysis (16s rRNA). Taxonomic nomenclature follows Hutterer (2005). Specimens belonging to problematic species complexes that are in need of revision were provisionally labelled with cheironyms, pending formal description.

### Step description

Field measurements

Sex and sexual condition were noted for each specimen:

- Males:
  - testes: abdominal
  - swelling of the epididymis: visible or not
- Females:
  - vagina: closed or perforated
  - nipples: small or swollen, lactating
  - pregnant: yes or no

Following measurements were taken from those specimens that were completely intact:

- Weight (in grams)
- Body length (head - tailbone, in mm)
- Length of tail (tail length in mm of the point of curvature (anus) until the tip of the tail)
- Size of the left hind leg (0.1 mm)
- Size of the left ear (0.1 mm)

Sample collection

Samples of liver, spleen and kidney were stored in 96% alcohol and RNA-later (only kidney). Blood samples were transferred to filter paper. Ectoparasites were preserved in 70% alcohol. Carcasses of specimens were stored at the Laboratory of Ecology and Animal Resource Management (University of Kisangani) and the Zoologisches Forschungsmuseum Alexander Koenig (Bonn). Tissues samples are stored at the Evolutionary Ecology Lab (University of Antwerp) and at the Royal Belgian Institute of Natural Sciences (Brussels). All specimens are stored under their field number.

For the collection in Yangambi, pictures were taken of each specimen's ventral, dorsal and lateral sides.

DNA Barcoding

DNA analysis of 16S-rRNA was conducted for a selection of individuals. For PCR amplification, we used the primer pair 16Sar-L (forward: 5′-CGCCTGTTTATCAAAAACAT-3′, Palumbi et al. 1991) and 16S-Hm (reverse: 5′AGATCACGTAGGACTTTAAT-3′, Quérouil et al. 2001). PCR amplification was performed in 15-µl reaction mixtures that contained 7.5 µl Qiagen Multiplex, 0.2 µM of each primer, 1.5 µl DNA template and 5.4 µl sterile deionised water. The reaction mixtures were preheated at 95°C for 15 min, followed by 42 amplification cycles (95°C for 30 s, 46°C for 90 s and 72°C for 90 s), with a final 10 min extension at 72°C. The samples were purified and sequenced in both directions at VIB Genetic Service Facility (University of Antwerp). Sequences were aligned using the Geneious software (Drummond et al. 2015).

## Geographic coverage

### Description

Lowland forests of the Kisangani, Isangi and Ubundu territories of the Tshopo province (former province Orientale), Democratic Republic of Congo.

### Coordinates

0°N and 1°N Latitude; 24°E and 27°E Longitude.

## Taxonomic coverage

### Description

All species belong to the family Soricidae, particularly the subfamily Crocidurinae. The dataset contains species from 5 genera: *Crocidura* (14 species), *Paracrocidura* (1), *Scutisorex* (2), *Suncus* (1) and *Sylvisorex* (4) (Table 2).

The dataset contains a number of specimens that likely belong to species new to science. Specimens morphologically resembling known species but found far outside the distribution of the known species have been identified using a cf. statement, others were named using a cheironym.

**Figure 2.**
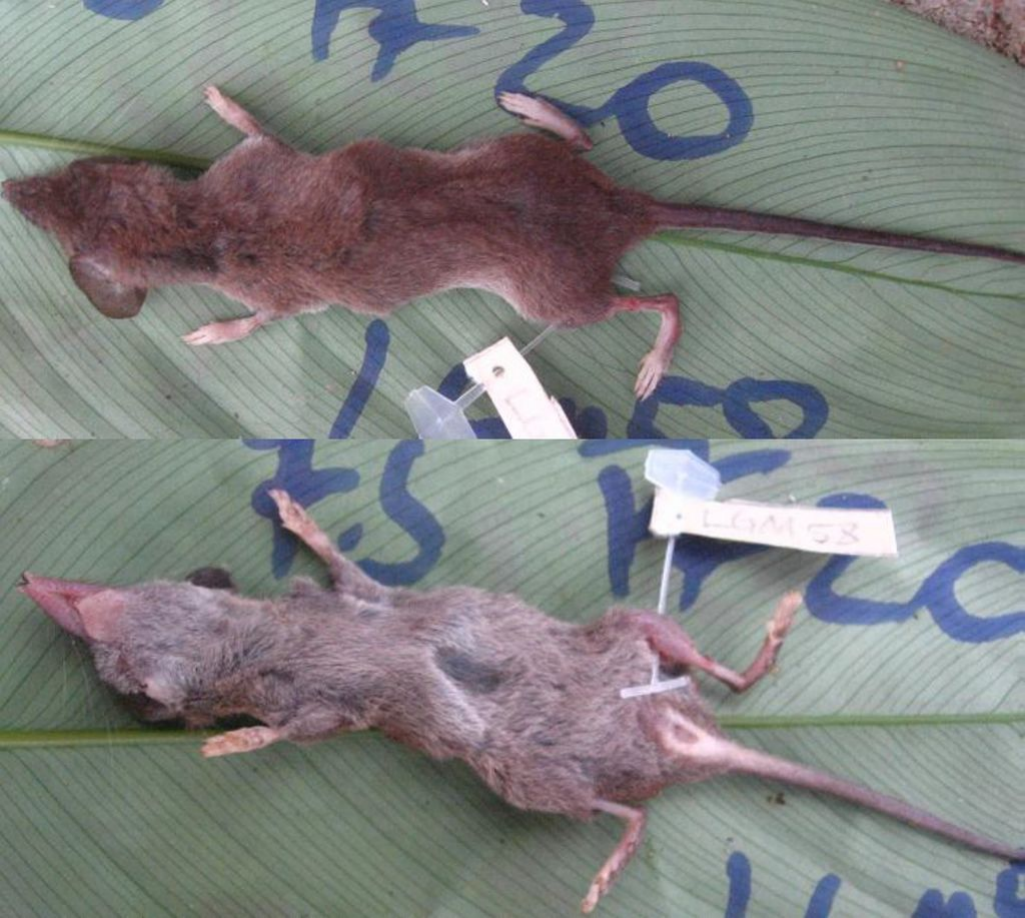
Dorsal and ventral view of *Crocidura sp1 yoko* (LEGM458, Mukinzi 2014).

*Crocidura sp1 yoko* has easily distinguishable characteristics: small size (4-6 g), brownish on the back, greyish-brown on the belly, brownish tail that is completely glabrous, except from the base which is covered with few small vibrissae, the down side of the tail clear, almost white at the base and around the anus and its small paws are equally light coloured (Fig. 2). Its skull resembles that of *Crocidura ludia* but is smaller (Mukinzi-Itoka 2014).

**Figure 3.**
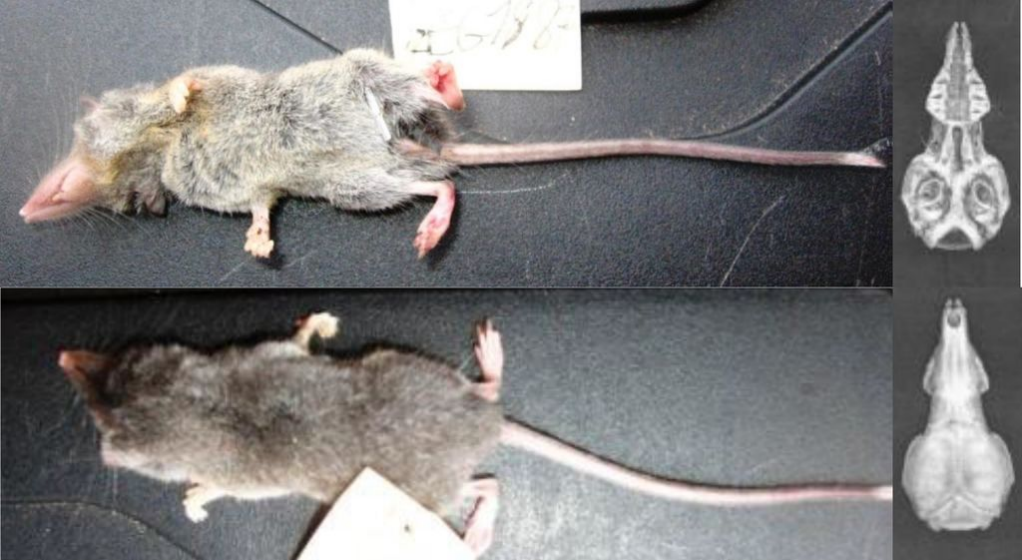
Dorsal and ventral view of body and skull of *Sylvisorex* n. sp. (LEGM1887, Mukinzi 2014).

*Sylvisorex* n.sp. is a small and rare species. Brown greyish on the back and silvery grey on the belly. It has a long tail covered with small hairs that grow longer and are more numerous towards the tip, forming a white brush. The tail is brown-black on top and slightly lighter on the bottom (Fig. 3). The species resembles *Crocidura polia* Hollister, 1916. However, the new species has little vibrissae on its tail while, in *C. polia*, half of the tail is covered with vibrissae (Mukinzi 2014).

*Scutisorex n. sp.* (description in progress, J. Hulselmans pers. comm.) was found in RF Yoko and its distribution seems to be limited to the forest bloc between the Lomami and Lualaba.

## Temporal coverage

### Notes

8 June 2005 (Yelenge) to 7 August 2014 (Yangambi).

## Collection data

### Collection name

Carcasses of specimens were stored at the Laboratory of Ecology and Animal Resource Management, University of Kisangani. Tissues samples are stored at the University of Antwerp and at the Royal Belgian Institute of Natural Sciences. For the collection of Yangambi, pictures were taken of each specimen's ventral, dorsal and lateral sides.

### Specimen preservation method

Samples of liver, spleen and kidney were stored in 96% alcohol and RNA-later (only kidney). Blood samples were transferred to filter paper. Ectoparasites were preserved in 70% alcohol. Specimen carcasses were stored in 70% alcohol.

## Usage rights

### Use license

Creative Commons Public Domain Waiver (CC-Zero)

## Data resources

### Data package title

African Mammalia

### Resource link

http://projects.biodiversity.be/africanmammalia

### Number of data sets

3

### Data set 1.

#### Data set name

Specimen list

#### Data format

.csv

#### Number of columns

35

#### Download URL

http://projects.biodiversity.be/africanmammalia/search

#### Description

The data can be downloaded from the online database, African Mammalia. Shrew specimens can be searched through the 'Search' or 'Taxa' tab.

**Data set 1.** 

Column label	Column description
Collection number	Museum collection number
Order	Taxonomic rank
Family	Taxonomic rank
Family author	Author(s) and publication date of family
Genus	Taxonomic rank
Genus author	Author(s) and publication date of genus
Species	Taxonomic rank
Species author	Author(s) and publication date of species
Subspecies	Taxonomic rank
Subspecies author	Author(s) and publication date of subspecies
Determinator	Determinator of specimen
Determination year	Determination year
Accuracy	Accuracy of determination
Field number	Unique identifier of specimen
Locality	Sampling locality
Altitude	Altitude of sampling location in metres
Altitude max	Maximum altitude
Country iso code	CD
Country	Democratic Republic Congo
Latitude	Latitude of sampling locality in decimal degrees
Longitude	Longitude of sampling locality in decimal degrees
Collector	Collector of specimen
Date collected	Date of collection
Date collected end	End of data collection
Sex	m, Male - f, Female
Sexual condition name	Sexual condition, see above
Sexual condition code	Sexual condition code
Age	Age of specimen
Weight	Weight in gram
Type	Holotype, paratype or syntype
Trap	Type of trap used
Available	Whether the specimen is present in the collection
Basis of record	Preserved specimen or observation
Tissues	Whether tissue samples are available
url	Link to the specimen information on African Mammalia

### Data set 2.

#### Data set name

Sequences

#### Data format

.csv

#### Number of columns

11

#### Download URL

http://projects.biodiversity.be/africanmammalia/search

#### Description

Export of DNA sequences and metadata.

**Data set 2.** 

Column label	Column description
Collection number	Museum collection number
Field number	Field number
Basis of record	Preserved specimen or observation
Family	Family, Soricidae
Genus	Genus name
Species	Species name
Subspecies	Subspecies name
Accession number	Genbank accession number
Sequence number	Unique sequence code
Sequence	DNA sequence
url	Link to the specimen information on African Mammalia.

### Data set 3.

#### Data set name

Measurements

#### Data format

.csv

#### Number of columns

14

#### Download URL

http://projects.biodiversity.be/africanmammalia/search

#### Description

Export of morphological measurements and metadata.

**Data set 3.** 

Column label	Column description
Collection number	Museum collection number
Field number	Unique field code
Basis of record	Preserved specimen or observation
Familiy	Family, Soricidae
Genus	Genus name
Species	Species name
Subspecies	Subspecies name
Sex	Sex (Male or Female)
hb	Head-body length
tl	Tail length
hf	Hind foot length
el	Ear length
m1-m25	Craniometric measurement, description available on http://projects.biodiversity.be/africanmammalia/about/data#measurements
url	Link to the specimen information on African Mammalia.

## Additional information

**Results communication**:

Results of diversity analyses have already been published in peer-reviewed journals (in chronological order):

- Mukinzi et al. (2005)
- Gambalemoke et al. (2008a)
- Gambalemoke et al. (2008b)
- Mukinzi et al. (2009)
- Van de Perre et al. (2018)

## Figures and Tables

**Table 1.** List of sampling sites including locality (and initials of collectors), coordinates, forest type (OG-X: Mixed old-growth forest; OG-M: Monodominant old-growth forest; RF: Regrowth forest; FL: Fallow land; OP: abandoned oil palm plantation), number of trapping sessions, start date of trapping and range of field numbers under which specimens are stored.

**Locality**	**Latitude**	**Longitude**	**Site**	**Forest type**	**Number of trapping sessions**	**Start date**	**Field numbers**
Baliko (SG)	0.6415	26.3639	Baliko_FP	OG-X	1	23/09/2006	BA77-675
	0.6415	26.3639	Baliko_FS	RF	1	23/09/2006	
	0.6415	26.3639	Baliko_JC	FL	1	23/09/2006	
Djabir (JCM, SG)	0.5192	24.1736	Djabir_FP_L1	OG-X	1	13/10/2005	DJ1-567
	0.5192	24.1736	Djabir_FP_L2	OG-X	1	13/10/2005	
	0.5192	24.1736	Djabir_FS	RF	1	13/10/2005	
Masako (JCM, SG)	0.6051	25.2565	Masako_FP	OG-X	1	2/06/2005	R27985-28242
	0.6051	25.2565	Masako_FS	RF	1	2/06/2005	
	0.6051	25.2565	Masako_FS_L1A	RF	1	12/03/2011	CRT3151-3520
	0.6051	25.2565	Masako_FS_L1C	RF	1	12/03/2011	
	0.6051	25.2565	Masako_Gil_L1A	OG-M	1	27/03/2012	MSK1-362
	0.6051	25.2565	Masako_Gil_L1C	OG-M	1	27/03/2012	
Yangambi (FVdP)	0.8144	24.4937	Yangambi_BRA1	OG-M	1	12/07/2015	COB2-1390
	0.7966	24.4978	Yangambi_GIL3	OG-M	1	8/05/2014	
	0.8081	24.5281	Yangambi_GIL4	OG-M	1	21/06/2013	
	0.7894	24.5175	Yangambi_JEU1	RF	1	20/06/2013	
	0.7949	24.4919	Yangambi_JEU2	RF	1	7/05/2014	
	0.7967	24.4941	Yangambi_JEU3	RF	1	13/07/2015	
	0.7931	24.4901	Yangambi_JEU4	RF	1	16/07/2016	
	0.7921	24.4972	Yangambi_JEU5	RF	1	17/07/2016	
	0.8135	24.5126	Yangambi_MIX2	OG-X	1	16/07/2016	
	0.7805	24.5211	Yangambi_MIX3	OG-X	1	20/06/2013	
	0.8144	24.4931	Yangambi_MIX5	OG-X	1	12/07/2015	
	0.8026	24.4875	Yangambi_MIX6	OG-X	1	7/05/2014	
Yelenge (JCM)	0.6387	25.0780	Yelenge_FP	OG-X	1	6/03/2005	R27622-27981
	0.6387	25.0780	Yelenge_FS	RF	1	6/03/2005	
Yoko (JCM)	0.2940	25.2881	Babogombe_FPG_L1	OG-M	5	21/04/2007	LEGM400-3017
	0.2940	25.2881	Babogombe_FPG_L2	OG-M	5	22/02/2007	
	0.2940	25.2881	Babogombe_FP_L1	OG-X	8	14/12/2006	
	0.2940	25.2881	Babogombe_FP_L2	OG-X	7	14/12/2006	
	0.2940	25.2881	Babogombe_FP_L3	OG-X	9	21/04/2007	
	0.2940	25.2881	Babogombe_FS_L1	RF	3	14/12/2006	
	0.2940	25.2881	Babogombe_FS_L2	RF	3	14/12/2006	
	0.2940	25.2881	Babogombe_JJ_L1	FL	3	15/12/2006	
	0.2940	25.2881	Babogombe_JV_L1	FL	3	23/02/2007	
	0.2940	25.2881	Babogombe_JV_L2	FL	2	15/12/2006	
	0.3234	25.2539	Kisesa_JJ	FL	8	18/10/2007	
	0.3234	25.2539	Kisesa_JV	FL	8	18/10/2007	
	0.3234	25.2539	Kisesa_VPS	OP	8	18/10/2007	

**Table 2.** Number of specimens per species caught in each locality.

**Species**	**Djabir**	**Yoko**	**Yangambi**	**Yelenge**	**Masako**	**Baliko**
*Crocidura caliginea* Hollister, 1916.	-	-	52	3	14	7
*Crocidura crenata* Brosset, Dubost & Heim de Balsac, 1965.	-	-	9	5	2	0
*Crocidura denti* Dollman, 1915.	-	-	104	8	25	11
*Crocidura dolichura* Peters, 1876.	13	111	8	6	5	0
*Crocidura cf. fuscomurina* Heuglin, 1865	-	4	-	-	-	-
*Crocidura goliath* Thomas, 1906.	0	10	-	-	-	-
*Crocidura grassei* Brosset, Dubost & Heim de Balsac, 1965.	0	26	-	-	-	-
*Crocidura latona* Hollister, 1916.	21	168	0	1	26	0
*Crocidura littoralis* Heller, 1910.	27	24	153	44	21	4
*Crocidura ludia* Hollister, 1916.	4	1013	27	0	34	11
*Crocidura cf. maurisca* Thomas, 1904	0	1	-	-	-	-
*Crocidura cf. muricauda* Miller, 1900	-	-	4	0	1	0
*Crocidura olivieri* Lesson, 1827.	6	173	43	0	11	10
*Crocidura yoko sp1*	0	64	-	-	-	-
*Paracrocidura schoutedeni* Heim de Balsac, 1956.	3	47	6	6	33	4
*Scutisorex congicus* Thomas, 1915.	-	-	4	1	22	6
*Scutisorex* n.sp.	-	75	-	-	-	-
*Suncus cf. remyi* Brosset, Dubost & Heim de Balsac, 1965.	-	-	27	0	5	5
*Sylvisorex akaibei* Mukinzi, Hutterer & Barriere, 2009.	-	-	27	1	0	1
*Sylvisorex cf. johnstoni* Dobson, 1888.	-	-	12	5	16	14
*Sylvisorex nsp1*	0	11	-	-	-	-
*Sylvisorex cf. ollula* Thomas, 1913.	6	275	-	-	-	-
Unidentified specimens	14	15	9	26	8	3
**Total**	**94**	**2017**	**485**	**106**	**223**	**76**
